# Estimating asymptomatic, undetected and total cases for the COVID-19 outbreak in Wuhan: a mathematical modeling study

**DOI:** 10.1186/s12879-021-06078-8

**Published:** 2021-05-25

**Authors:** Xi Huo, Jing Chen, Shigui Ruan

**Affiliations:** 1 Department of Mathematics, University of Miami, 1365 Memorial Drive, Coral Gables, FL, 33146 USA; 2 Department of Mathematics, Nova Southeastern University, 3301 College Ave, Fort Lauderdale, FL, 33314 USA; 3 Sylvester Comprehensive Cancer Center, University of Miami Miller School of Medicine, 1475 NW 12th Ave, Miami, FL, 33316 USA

**Keywords:** COVID-19, Wuhan, Asymptomatic cases, Undetected cases, Total number of infections, Mathematical modeling

## Abstract

**Background:**

The COVID-19 outbreak in Wuhan started in December 2019 and was under control by the end of March 2020 with a total of 50,006 confirmed cases by the implementation of a series of nonpharmaceutical interventions (NPIs) including unprecedented lockdown of the city. This study analyzes the complete outbreak data from Wuhan, assesses the impact of these public health interventions, and estimates the asymptomatic, undetected and total cases for the COVID-19 outbreak in Wuhan.

**Methods:**

By taking different stages of the outbreak into account, we developed a time-dependent compartmental model to describe the dynamics of disease transmission and case detection and reporting. Model coefficients were parameterized by using the reported cases and following key events and escalated control strategies. Then the model was used to calibrate the complete outbreak data by using the Monte Carlo Markov Chain (MCMC) method. Finally we used the model to estimate asymptomatic and undetected cases and approximate the overall antibody prevalence level.

**Results:**

We found that the transmission rate between Jan 24 and Feb 1, 2020, was twice as large as that before the lockdown on Jan 23 and 67.6*%* (95% CI [0.584,0.759]) of detectable infections occurred during this period. Based on the reported estimates that around 20% of infections were asymptomatic and their transmission ability was about 70% of symptomatic ones, we estimated that there were about 14,448 asymptomatic and undetected cases (95% CI [12,364,23,254]), which yields an estimate of a total of 64,454 infected cases (95% CI [62,370,73,260]), and the overall antibody prevalence level in the population of Wuhan was 0.745% (95% CI [0.693*%*,0.814*%*]) by March 31, 2020.

**Conclusions:**

We conclude that the control of the COVID-19 outbreak in Wuhan was achieved via the enforcement of a combination of multiple NPIs: the lockdown on Jan 23, the stay-at-home order on Feb 2, the massive isolation of all symptomatic individuals via newly constructed special shelter hospitals on Feb 6, and the large scale screening process on Feb 18. Our results indicate that the population in Wuhan is far away from establishing herd immunity and provide insights for other affected countries and regions in designing control strategies and planing vaccination programs.

## Background

On December 31, 2019, a cluster of 27 cases of pneumonia of unknown etiology were detected in Wuhan, Hubei Province, China [1–5]. As all of the first set of 27 infected patients were associated with a seafood and wild animal market and the virus was found in the market, it is believed that the virus very likely came from wild animals [3–5]. On January 10, 2020, the number of cases increased to 41 with six serious cases and one disease-induced death [3, 5–7]. Detailed clinic features of these 41 patients were reported two weeks later [3]. The sequence of the agent’s RNA genome was determined and it was identified as a betacoronavirus [8]. Consequently, the virus was named *Severe Acute Respiratory Syndrome Coronavirus 2* (SARS-CoV-2) and the disease caused by the virus was named *Coronavirus Disease in 2019* (COVID-19) on February 11, 2020 [9]. As Wuhan is a crucial provincial, national, and international travel hub located in Central China with 11 million residents plus 3 million nonresidents (“floating population”) and Chunyun (Spring Festival travel season, Jan 10 to Feb 18) had already started, the virus spread rapidly from Wuhan to all 13 prefectures in Hubei Province as well as all other 32 provinces, autonomous regions, municipalities, and special administrative regions in China by mid-February [10]. The virus had also been spread to many countries and territories and on March 11, 2020, WHO declared COVID-19 a global pandemic [9]. By the end of August 2020, it had been reported in 216 countries and regions worldwide with more than 25 million infected cases including more than 800,000 deaths [9].

**Fig. 1:**
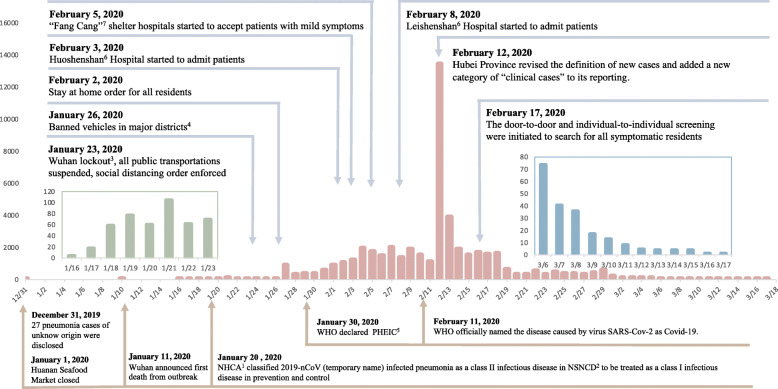
Daily reported cases, important events and timelines in Wuhan from Dec 8, 2019 to March 18, 2020, obtained from Wuhan Municipal Health Commission website [18]. 1. NHCC - National Health Commission of China; 2. NSNCD - National Stationary Notifiable Communicable Diseases; 3. Jan 24, starting at 10:00am, Wuhan suspended all means of public transport (buses, ferries, subways, etc.) in the city; closed all outbound routes via buses, flights and railways; closed Yangtze River Tunnel; 4. Jan 26, Wuhan banned all motor vehicles in the city center; 5. WHO declared a novel coronavirus outbreak that originated in Wuhan a public health emergency of international concern (PHEIC); 6. Huoshenshan and Leishenshan hospitals were two newly built ad hoc hospitals with 1,000 beds and 1,600 beds, respectively, and facilities designed to treat COVID-19 patients; 7. “Fangcang” shelter hospitals: Wuhan turned 11 sports centers, exhibition halls, and other local venues into makeshift hospitals with more than 10,000 beds for confirmed COVID-19 patients with mild symptoms

In the early stage, local authorities in Wuhan took several measures to combat the spread of the coronavirus, including closing the seafood market, treating the infected individuals in a designated hospital, tracing those who had contact with the infected patients and putting them in quarantine or under medical observation, and so on. On Jan 20, 2020, the National Health Commission of China (NHCC) classified novel coronavirus infected pneumonia as a class II infectious disease in the National Stationary Notifiable Communicable Diseases (NSNCD) to be treated as a class I infectious disease in prevention and control (Fig. 1) [11]. On Jan 23, 2020, in order to control local outbreak and prevent further exportation to other regions, the Wuhan Municipal authority locked down the entire city and suspended all local (bus, ferry, subway) and long-distance (bus, train and flight) public transportation [12]. Social distance policy (staying at home and wearing face masks in public) was also implemented. During the two weeks after lockdown, the number of cases increased significantly when Wuhan was facing a severe shortage of medical resources, including health care workers, hospital beds, personal protective equipments (PPEs), and testing facilities. To overcome these difficulties, a number of hospitals were turned into specialty hospitals to treat COVID-19 patients, two emergency specialty hospitals were constructed timely and speedily, and the novel idea of Fangcang shelter hospital was initiated with several of them quickly developed in days (Fig. 1) [13]. Meanwhile, starting from Jan 24, 2020, 346 medical teams with more than 42,600 medical workers from across China were sent to Hubei Province to help fight the virus, among them more than 35,000 were dispatched to Wuhan which doubled the medical manpower in the city [14]. These improvements of medical resources enabled the implementation of the centralized quarantine and treatment for all confirmed and presumptive cases, which also effectively helped with the isolation of the ills from their family members and communities. Starting from Feb 17, 2020, a large scale door-to-door and individual-to-individual screening policy had been conducted for all residents, by doing so all symptomatic individuals were identified and isolated during this phase [15, 16]. From March 18 to March 31, there were no more new symptomatic cases reported and the first wave of COVID-19 outbreak in Wuhan was successfully controlled with a total of 50,006 reported symptomatic cases [17]. On April 8, 2020, the lockdown of Wuhan was officially lifted. Meanwhile, Wuhan Municipal Health Commission (WMHC) started to report asymptomatic cases on April 1, 2020, on a daily basis and a total of 1,173 asymptomatic cases were reported from April 1 to May 31 when the last symptomatic cases were identified [18].

Mathematical modeling has become an important and useful tool in analyzing the epidemiological characteristics of infectious diseases. The scientific community responded to the outbreak of COVID-19 in Wuhan very promptly and efficiently with a number of modeling studies published based on the early outbreak data in and exported from Wuhan. The early modeling studies have greatly helped policy makers in understanding the epidemiological characteristics of COVID-19 [4, 19], assessing the speed of spatial transmission [20–22], predicting possible outcomes of the outbreak [23–26], and evaluating efficacy of various nonpharmaceutical intervention strategies (NPIs) [27–31]. In particular, a dataset of 32,583 laboratory confirmed cases was analyzed [15] by a well-developed statistical method [32] which requires the date of symptom onset for each patient - a piece of information not publicly available for all reported cases in Wuhan. The analysis [15] focused on calculating the time-varying effective reproduction number *R*_*t*_ and the time point for *R*_*t*_ falling below 1 was believed as when the nonpharmaceutical intervention became completely effective [33]. Based on the same dataset of the 32,583 laboratory confirmed cases, a modeling approach was used [16] to reconstruct the full-spectrum dynamics of COVID-19 between Jan 1 and March 8, 2020 across five periods marked by events and interventions.

Note that the number of officially reported COVID-19 cases (clinically diagnosed and laboratory confirmed) in Wuhan was 50,006 by the end of March 2020 [17], which has not been studied in the literature, neither by statistical analysis nor by mathematical modeling. Moreover, the dynamics of asymptomatic cases, the impact of these asymptomatic cases on the transmission dynamics, and the possibility of undetected cases [34] have not been thoroughly investigated for the COVID-19 outbreak in Wuhan based on the complete reported data.

**Fig. 2:**
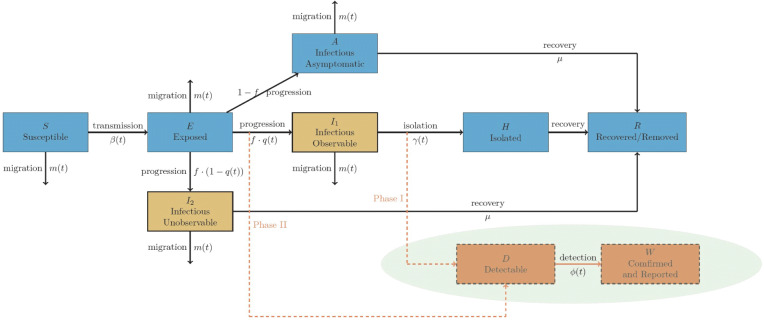
Transmission and detection dynamics of COVID-19 in Wuhan. Compartments in blank background describe the transmission dynamics while compartments in mint background refer to the case detection and reporting dynamics. The population is stratified into seven compartments: susceptible (*S*(*t*)), exposed (*E*(*t*)), infectious but asymptomatic (*A*(*t*)), infectious, symptomatic, and detectable (*I*_1_(*t*)), infectious, symptomatic, but undetectable (*I*_2_(*t*)), effectively isolated (*H*(*t*)), recovered or removed due to death (*R*(*t*)) at time *t*. For the dynamics of case identification and reporting, *D*(*t*) is the number of cases that are detectable but not yet detected or reported and *W*(*t*) is the number of cases that have been reported at time *t*. Phase I represents the period before Jan 23 (the day of lockdown) and Phase II refers to the period after Jan 23

In this paper, we developed a compartmental model (Fig. 2) to describe the dynamics of disease transmission and case identification of COVID-19 in Wuhan, parameterized the time-dependent model coefficients based on the reported data and well-documented timelines on controlling COVID-19 in Wuhan (Fig. 1) [13, 15, 16, 35, 36], and used the model to calibrate the 50,006 reported cases by the end of March 2020. Our goals were to use data fitting results to infer the average strength of the nonpharmaceutical intervention strategies during each stage of the outbreak, estimate the scale of unobserved symptomatic cases, project the number of infections in different stage of the outbreak from the hidden dynamics, and calculate the overall attack ratio, that is the antibody prevalence level in the population, based on various assumptions on the percentage and infectiousness of the asymptomatic cases.

## Methods

Our model simulations were used to capture the dynamics of COVID-19 transmission, case detection and report during several time periods corresponding to different levels of public health interventions implemented in Wuhan [15, 16]. Before the lockdown on Jan 23, 2020, no strong interventions were imposed. Between Jan 24 and Feb 1, 2020, social distancing measures were first implemented, and many infected individuals were not diagnosed and were only self-isolated at home due to the shortage of medical resources. On Feb 2, 2020, all residents were required to stay at home, and beginning from Feb 6, 2020, makeshift hospitals were set up and started to admit large number of patients, mostly with mild symptoms [13]. On Feb 17, 2020, large scale door-to-door and individual-to-individual screening was initiated to identify for all symptomatic residents (Fig. 1).

In our simulations, the transmission rate varied in the three periods with escalated restrictions on activities of local residents: no intervention, social distancing order, and mandatory stay-at-home. The isolation rate changed in the three periods with varied medical resources: before lockdown when the hospitals were not overwhelmed, post lockdown when the makeshift hospitals were still under construction, and two weeks after lockdown when Fangcang hospitals started admitting patients. Asymptomatic (subclinical) individuals were unlikely to be detected, while some symptomatic individuals could also be undetected due to the scarcity of testing facilities and public awareness. The fraction of detectable symptomatic individuals would vary in three periods: before Jan 23, 2020 when not all symptomatic cases would seek for medical diagnosis, between Jan 24 and Feb 17, 2020 when testing resources were insufficient, and after Feb 17, 2020 when no symptomatic cases would be missed because of the massive population-wide screening. The average delay from symptom onset to confirmation and report varied throughout five periods of the outbreak, and the rate of reporting in our model was parameterized accordingly. The mass population migration during Chunyun was modeled by an emigration rate during the two-week window right before lockdown. Further, the infectiousness stage within which symptomatic individuals can be detected would differ before and after the lockdown due to public awareness: before the lockdown only those who were hospitalized could be tested and diagnosed, while after the lockdown any individual with onset symptom could seek for diagnosis and become detectable.

### The time-dependent transmission model

We developed a time-dependent deterministic model to simulate the transmission dynamics of COVID-19 and the detection-report dynamics of identifiable cases, with time-dependent parameters and terms reflecting the variations of NPIs and detection capacities during different stages of the outbreak. The population in Wuhan is stratified into seven compartments at any time *t*: susceptible (*S*), exposed (*E*), infectious but asymptomatic (*A*), infectious, symptomatic, and detectable (*I*_1_), infectious, symptomatic, but undetectable (*I*_2_), effectively isolated (*H*), recovered or removed due to death (*R*). This classification is based on various assumptions. Firstly, considerable evidence suggested that there existed asymptomatic yet infectious individuals, so we assumed that a fixed proportion *f* of the infectious population would develop symptoms, the rest of them would be asymptomatic and hence would not be detected at all. Secondly, as the testing and detection abilities in Wuhan kept evolving during the outbreak, it is highly possible that only a fraction of symptomatic cases could be observed and reported. Thus we assumed that among those symptomatic individuals, a proportion *q*(*t*) of them would be observed, tested (or diagnosed), then reported as confirmed cases, while the rest of them would not be observed. Further, we assumed that the incubation period overlaps with latent period and with an average length of 5.2 days [37]. Thus the exposed individuals are neither symptomatic nor infectious.

In addition, we incorporated the dynamics of case identification and reporting in the model. In Wuhan, each case was first detected (either via RT-PCR lab test or via clinical diagnosis) and then reported as a confirmed case. We denoted *D*(*t*) as the number of cases that were detectable but not yet detected or reported at time *t*, and *W*(*t*) as the number of cases that had been reported at time *t*. Therefore, *W*(*t*) corresponds to the cumulative number of reported cases at time *t*, while the inflow from *D*(*t*) to *W*(*t*) each day would correspond to the daily reported cases. Note that the case identification and reporting dynamics were derived to keep track of the case counts, not the actual population. Thus the equations about *D*(*t*) and *W*(*t*) were decoupled from the transmission dynamics and were only used for data fitting. The compartmental dynamics are illustrated in Fig. 2 and model is described by the following time-dependent ordinary differential equations:

1 $$\begin{array}{@{}rcl@{}} \begin{aligned} {\frac{dS(t) }{dt}=-\beta(t) S(t) \frac{I_{1}(t)+I_{2}(t)+pA(t)}{N(t)} - m(t) S(t),} \\ {\frac{dE(t) }{dt}=\beta(t) S(t) \frac{I_{1}(t)+I_{2}(t)+pA(t)}{N(t)}-\sigma E(t) - m(t) E(t),} \\ {\frac{dI_{1}(t) }{dt}=q(t) \cdot f\cdot \sigma E(t)- \gamma(t) I_{1}(t) - m(t) I_{1}(t),} \\ {\frac{dI_{2}(t) }{dt}=(1-q(t))\cdot f \cdot \sigma E(t)- \mu I_{2}(t) - m(t) I_{2}(t),} \\ {\frac{dA(t) }{dt}=(1-f)\cdot \sigma E(t)-\mu A(t) - m(t) A(t),} \\ {\frac{dD(t) }{dt}=\lambda(t) - \phi(t) D(t),} \\ {\frac{dW(t) }{dt}=\phi(t) D(t),} \\ \lambda(t)= \left\{ \begin{array}{ll} \gamma(t) I_{1}(t),\,\,t\le \text{January 23, 2020 (Phase I)},\\ q(t) f \sigma E(t),\,\,t> \text{January 23, 2020 (Phase II)}. \end{array} \right. \end{aligned}  \end{array} $$

As shown in model (1), the inflow to *D* varies in different phases: we assumed that symptomatic and detectable cases can only be tested and reported after hospitalization before the lockdown as there was a lack of public awareness and test availability; and can be tested and reported upon symptom onsets after the lockdown because of the population-wide alertness about the virus and expanded test capacity. Therefore, *ϕ*(*t*) represents the rate from hospitalization to report for the time before the lockdown, and represents the rate from onset of symptom to report for the time after the lockdown. The time-dependent parameters were assumed as step functions, where the cutoff date for each stage was retrieved from various literature and news reports [13, 15, 16, 37, 38].

- The transmission rate *β*(*t*) can be expressed as a product of the overall population contact rate and the probability that a contagion incidence happens during each contact, where the value of *β*(*t*) at each stage represents the effects of NPIs including mass quarantine, social distancing, use of face masks, and use of PPEs in health care workers. We respectively assumed a constant transmission rate *β*_1_ on and before Jan 23, 2020, *β*_2_ from Jan 24, 2020 to Feb 1, 2020, and *β*_3_ on and after Feb 2, 2020. Transmission rates at all stages were estimated from data fitting.
  $${}\begin{aligned} \beta(t)= \left\{ \begin{array}{lll} \beta_{1},\,\,t\le \text{January 23, 2020},\\ \beta_{2},\,\,\text{January 23, 2020}< t\le \text{February 1, 2020},\\ \beta_{3},\,\,t>\text{February 1, 2020}. \end{array} \right. \end{aligned} $$
- Isolation rate *γ*(*t*) of symptomatic individuals was determined directly by the capacities of hospital beds and isolation facilities, which were of severe shortage after the lockdown and then had increased fourfold as two new hospitals and several Fangcang shelter hospitals were built in a short time. We assumed a constant isolation rate *γ*_1_ on and before Jan 23, 2020, *γ*_2_ from Jan 24, 2020 to Feb 6, 2020, and *γ*_3_ on and after Feb 7, 2020 when Fangcang shelter hospitals started admitting patients. We adopted information from the early stage of the outbreak [37] and fixed *γ*_1_=1/9.1 day^−1^. Both *γ*_2_ and *γ*_3_ were hard to be estimated due to limited hospital beds information, but estimating both from data fitting would result in parameter unidentifiability issues, so we fixed *γ*_2_ at various values (1/3, 1/6, 1/9, 1/12), and estimated all other unknown parameters including *γ*_3_ in multiple scenarios.
  $${}\begin{aligned} \gamma(t)= \left\{ \begin{array}{lll} \gamma_{1},\,\,t\le \text{January 23, 2020},\\ \gamma_{2},\,\,\text{January 23, 2020}< t\le \text{February 6, 2020},\\ \gamma_{3},\,\,t>\text{February 6, 2020}. \end{array} \right. \end{aligned} $$
- The fraction *q*(*t*) of observable cases would vary with respect to public awareness, surveillance intensity and testing abilities, where in Wuhan there was a low public awareness of the emerging outbreak before the lockdown, while a massive community screening for symptomatic individuals was launched around Feb 19, 2020. We assumed a fraction *q*_1_ of symptomatic cases were detectable on and before Jan 23, 2020, a fraction *q*_2_ of symptomatic cases were detectable during Jan 24 and Feb 18, 2020, and all symptomatic cases were detectable after Feb 19, 2020. Both *q*_1_ and *q*_2_ were estimated from data fitting.
  $${}\begin{aligned} q(t)= \left\{ \begin{array}{lll} q_{1},\,\,t\le \text{January 23, 2020},\\ q_{2},\,\,\text{January 23, 2020}< t\le \text{February 17, 2020},\\ q_{3},\,\,t>\text{February 17, 2020}. \end{array} \right. \end{aligned} $$
- There were notable delays between symptom onset and laboratory confirmation throughout all stages of the outbreak, where a detailed statistics for all patients in Wuhan has been well-documented [15, 16]. We can therefore calculate the mean values of the delays in these five periods: 23 days on and before Jan 10, 15 days from Jan 11 to Jan 23, 11 days from Jan 24 to Feb 1, 7 days from Feb 2 to Feb 16, and 4 days after Feb 17, 2020. In this way, the case detection and report rate *ϕ*(*t*) can be parameterized accordingly. In particular, during the first two stages, we assumed that patients were only detectable after hospitalization, given the average onset to hospitalization period as 9.1 days [37], we thus had the delay between hospitalization to detection to be 13.9 and 5.9 days respectively in the two periods prior to the lockdown.
  $${}\begin{aligned} \phi(t)= \left\{ \begin{array}{lllll} \phi_{0},\,\,t\le \text{January 10, 2020},\\ \phi_{1},\,\,\text{January 10, 2020}< t\le \text{January 23, 2020},\\ \phi_{2},\,\,\text{January 23, 2020}< t\le \text{February 1, 2020},\\ \phi_{3},\,\,\text{February 1, 2020}< t\le \text{February 16, 2020},\\ \phi_{4},\,\,t>\text{February 16, 2020}. \end{array} \right. \end{aligned} $$
- Chunyun is the busiest travel season in China which began on Jan 10 in 2020. It was reported that 5 million people had already left Wuhan in this period, leaving 9 million local population under lockdown and massive quarantine [38]. We used a linear net migration rate *m*(*t*) to model the massive population emigration from Wuhan: fixed the total population in Wuhan before Jan 10, 2020 as 14 million, and the total population after Jan 23, 2020 as 9 million, then set *m*(*t*)=0.03155 day^−1^ between Jan 10 and Jan 23, 2020 so that the total population can gradually decrease from 14 million to 9 million during a two-week window. In particular, we assumed that isolated individuals were not mobile and all other population compartments were modeled with the net emigration rate.
  $${}\begin{aligned} m(t)= \left\{ \begin{array}{lll} 0,\,\, t<\text{January 10, 2020},\\ m,\,\,\text{January 10, 2020}\le t\le \text{January 23, 2020},\\ 0,\,\,t>\text{January 23, 2020}. \end{array} \right. \end{aligned} $$

### Data collection

We searched the websites of the local Wuhan Municipal Health Commission (WMHC) (http://wjw.wuhan.gov.cn/), Health Commission of Hubei Province (HCHP) (http://wjw.hubei.gov.cn/), National Health Commission of China (NHCC), http://www.nhc.gov.cn/, as well as World Health Organization (WHO), https://www.who.int/ in both Chinese and English for data, extracted the local case counts in Wuhan, and obtained a data set up to March 31, 2020. The symptom onset of the first confirmed case can be dated back to Dec 8, 2019, and the first case cluster was included in the set of probable case count of 27 reported as early as Dec 31, 2019 [1, 3, 5]. We thus incorporated the 27 case count as the first data point. The total of 41 cases reported on Jan 10, 2020 was our second data point [3, 5, 6]. Starting from Jan 11, 2020, WMHC has been providing a daily confirmed case report for Wuhan City, and starting from Jan 22, 2020, HCHP has been giving a daily briefing on the outbreak data for Hubei Province that includes data for Wuhan. Therefore, we used the confirmed cases (both laboratory confirmed and clinically diagnosed) that were reported on Dec 31, 2019 and continuously reported from Jan 10 to March 31, 2020 for our fitting [18]. Note that there were 50,007 reported cases on March 31, 2020 [17], but one of the cases was an imported case. As we were studying the local outbreak in Wuhan, we excluded the imported case in our simulations.

### Fitting data

We conducted multiple fitting experiments under various assumptions on the asymptomatic individuals. There is a wide range of estimates on the fraction of symptomatic cases (*f*) and their reduced transmission ability (*p*) [39–42]. Here we picked a total of 81 possible pairs of (*f*,*p*) ranging from 0.1∼0.9 for each parameter. Then for each pair of (*f*,*p*), we performed data fitting in two separate phases and compared the goodness-of-fit by estimating elpd_loo_ - the expected log pointwise predictive density using leave-one-out (loo) cross validation [43, 44].

**Phase I:** Cumulative reported case data from Dec 31, 2019 to Jan 23, 2020 were fitted to our model via the Monte Carlo Markov Chain (MCMC) method by using the software Stan [43]. Specifically, the model was initiated on Dec 8, 2019 (day 0) with 14 million susceptible individuals, 1 symptomatic case, and zero for all other compartments. We estimated the values of the transmission rate (*β*_1_) and the fraction of detectable cases (*q*_1_), with uniform prior distributions in (0,5) and (0,1) respectively. For the likelihood function, we assumed that the cumulative observed cases on day *t*, *X*_*t*_, follows a lognormal distribution with mean given by ln*W*(*t*) from the model; that is,

$$X_{t} \sim \text{LogNormal}(\ln{W(t)},\sigma_{0}^{2}),$$

where *σ*_0_>0 was sampled together with the estimated parameters. Convergence was checked by calculating the $\hat {R}$ value in Gelman-Rubin diagnostic [45] and examining the effective sample size. Phase I corresponds to the exponential growth of the epidemic, and the predicted values for model compartments all vary in wide bands. To continue our fitting for the next phase, we picked the median value for each compartment predicted from the model and set them as the initial condition for Jan 23, 2020 (day 46) so as to initiate the next stage fitting.

**Phase II:** We fitted cumulative reported case data from Jan 24 to Mar 31, 2020 to our model via the same techniques in Phase I. Preliminary experiments showed that the five essential parameters, *β*_2_,*β*_3_,*q*_2_,*γ*_2_,*γ*_3_ were inter-dependent and fitting all of them to the data would result in parameter unidentifiability issues. Therefore, in order to achieve credible fitting results one has to fix one more parameter from the five unknowns. We chose to fix the isolation rate between Jan 24 to Feb 6 (*γ*_2_) at different values in comparison to the isolation rate before lockdown (*γ*_1_). Between Jan 24 and Feb 6, 2020, the unprecedented lockdown was suddenly enforced and medical resources were scarce, and it was unclear if the actual isolation rate during this period was faster or slower than that before the lockdown: on one hand, this period was reported to be the most difficult period for symptomatic individuals to seek for health cares [15, 16] which could delay hospitalizations; on the other hand, this was also a period with rapidly enhanced public alertness of the emerging pathogen which could lead to voluntary self-isolation. So we made several hypothesized scenarios by fixing *γ*_2_ at various values and then fitted the other 4 parameters to data. Specifically, we let *γ*_2_=1/3, 1/6, 1/9, 1/12, and named the corresponding fittings respectively as **3-day, 6-day, 9-day, and 12-day isolation scenario.** Each scenario corresponds to an assumed comparison between the overall isolation rate from Jan 24 to Feb 6 and the isolation rate before lockdown: 3-day and 6-day scenarios assumed faster isolation after lockdown, 9-day scenario assumed similar isolation before and after lockdown, while 12-day scenario assumed slower isolation after lockdown.

## Results

We conducted the two-phase fitting with a total of 324 times with parameter set (*f*,*p*,*γ*_2_) fixed at various presumable values. The description on the fixed, varied, and fitted parameters were summarized in Table 1.

**Table 1:** Table of Parameters

Parameter	Description	Value	Resources
*β*_1_	transmission rate on and before 1/23/2020	Fitted	-
*β*_2_	transmission rate from 1/24/2020 to 2/1/2020	Fitted	-
*β*_3_	transmission rate on and after 2/2/2020	Fitted	-
*γ*_1_	isolation rate on and before 1/23/2020	1/9.1 day^−1^	[37]
*γ*_2_	isolation rate from 1/24/2020 to 2/6/2020	Varied	[13]
*γ*_3_	isolation rate on and after 2/7/2020	Fitted	-
*q*_1_	fraction of observable cases on and before 1/23/2020	Fitted	-
*q*_2_	fraction of observable cases from 1/24/2020 to 2/17/2020	Fitted	-
*q*_3_	fraction of observable cases on and after 2/18/2020	1.0	[15, 35]
*ϕ*_0_	detection and report rate on and before 1/10/2020	1/13.9 day^−1^	[15]
*ϕ*_1_	detection and report rate from 1/11/2020 to 1/23/2020	1/5.9 day^−1^	[15]
*ϕ*_2_	detection and report rate from 1/24/2020 to 2/2/2020	1/11 day^−1^	[15]
*ϕ*_3_	detection and report rate from 2/3/2020 to 2/16/2020	1/7 day^−1^	[15]
*ϕ*_4_	detection and report rate on and after 2/17/2020	1/4 day^−1^	[15]
*μ*	recovery rate	1/14 day^−1^	[42]
*σ*	infectiousness development rate	1/5.2 day^−1^	[37, 46]
*f*	fraction of symptomatic cases	Varied	[39–41]
*p*	reduced transmissibility of asymptomatic individuals	Varied	[35, 39, 41]
*m*	net population migration rate from 1/10/2020 to 1/23/2020	0.03155 day^−1^	Calculated
*N*_1_	total population in Wuhan on and before 1/23/2020	14 million	[38]
*N*_2_	total population in Wuhan on and after 1/24/2020	9 million	[38]

### Fitting outcomes

**Fig. 3:**
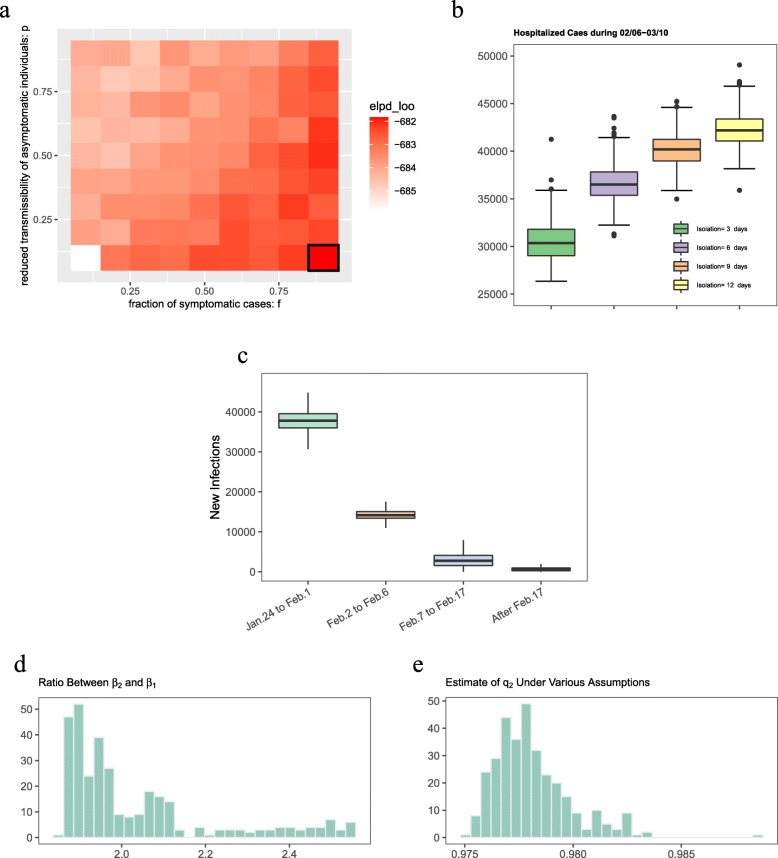
**a** Goodness of fit for simulations under various assumptions on (*f*,*p*) and with $\gamma _{2}^{-1}=3\,\text {days}$. **b** Numbers of patients isolated from Feb 6 to Mar 10, 2020 - the operation period of Fangcang shelter hospitals. The simulations were done based on *f*=0.9, *p*=0.1 and with various *γ*_2_ values. **c** Numbers of new infections in various phases. Simulations were carried out based on $f=0.9,\,p=0.1,\,\gamma _{2}^{-1}=3\,\text {days}$. **d** Ratios between *β*_2_ and *β*_1_ under all assumptions. The plot represents the distribution of a total of 324 ratios $\overline {\beta _{2}}/\overline {\beta _{1}}$, where $\overline {\beta _{1}}$ ($\overline {\beta _{2}}$) is the posterior median of *β*_1_ (*β*_2_) from Phase I (Phase II) fitting under each combination of (*f*,*p*,*γ*_2_). **e** Posterior medians of *q*_2_ under all assumptions. The plot shows the distribution of the posterior medians of *q*_2_ from Phase II fitting under all combinations of (*f*,*p*,*γ*_2_)

**Fig. 4:**
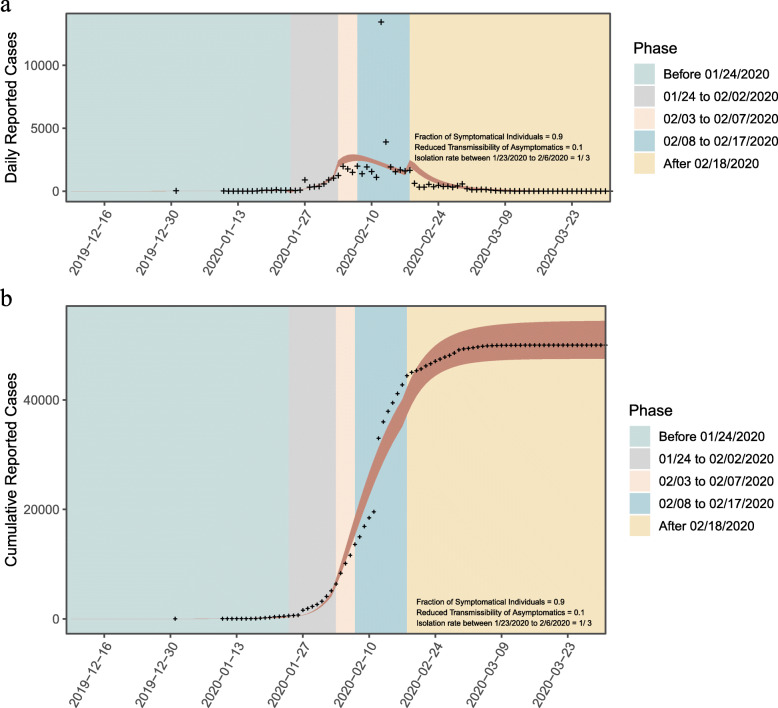
Simulations of the reported COVID-19 cases in Wuhan from Dec 8, 2019 to March 31, 2020, by using our model. **a** Simulation of the daily reported cases; **b** Simulation of the cumulative COVID-19 cases. The simulations were performed based on $f=0.9,\,p=0.1,\,\gamma _{2}^{-1}=3\,\text {days}$

Firstly, we compared the goodness of fit for each parameter set by evaluating the expected log pointwise predictive density using the LOO package in R [43, 44] and plotted the value for each fitting in four heatmaps about (*f*,*p*) with *γ*_2_ respectively fixed at its four assumed values. Figure 3a shows one heatmap with *γ*_2_=1/3 where the set (0.9,0.1,1/3) circled in black has the largest elpd_loo_ value among all 324 fittings. However, the standard errors of elpd_loo_ for all fittings share similar values around 8.3, which was of the same scale with the biggest difference among all elpd_loo_ values. This indicates that there is no big difference between all 324 fittings and there is also no best fitting scenario under which one can select the most possible values of *f*,*p*, and *γ*_2_. The fitting results were shown in Fig. 4 for the parameter set (0.9,0.1,1/3) as a representative, while the results from all other fittings are visually similar.

**Fig. 5:**
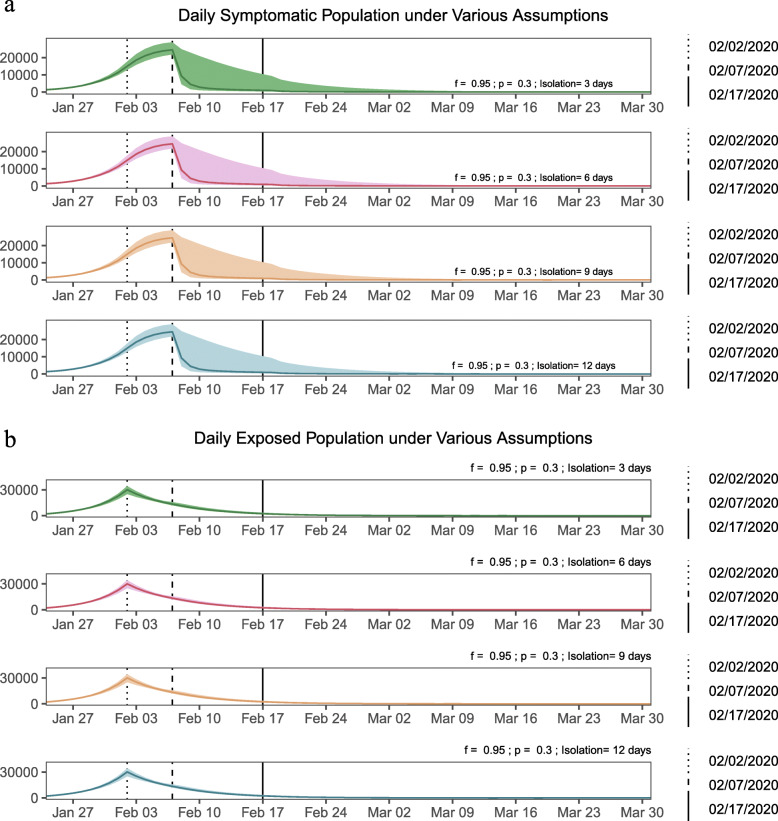
Daily exposed and unisolated symptomatic populations. The reconstructed transmission dynamics about exposed and infectious populations are not dependent on the parameter set used for fitting. We observed that the exposed population peaked on Feb 2, 2020, and the unisolated symptomatic individuals peaked on Feb 6, 2020

Our model was used to calibrate the reported COVID-19 cases in Wuhan from Dec 8, 2019 to March 31, 2020 (Fig. 4) and to explain the sudden spike of confirmed cases on Feb 12, 2020, when 14,840 new cases (including 13,332 clinically diagnosed cases) were reported in a single day. The key was to take into consideration the phased intervention strategies, health care resources, and more importantly the delay from symptom onset to diagnosis and report. Further, some hidden dynamics of the transmission such as the daily exposed and infectious populations can be simulated via the well-parameterized model. We found that, regardless of the presumed parameter set, under all scenarios the exposed population peaked on Feb 2, 2020 - right before the stay-at-home order was enforced, and the unisolated symptomatic individuals peaked on Feb 6, 2020 - right before the Fangcang shelter hospitals started to admit a large number of patients (Fig. 5).

Figure 3b shows that the total number of isolated cases during the operation period of Fangcang shelter hospitals would differ in terms of the assumed value for *γ*_2_: the more effective the isolation was before Feb 6, 2020, the less patients were left for admission to the newly constructed health care facilities. Additionally, this measurement was not sensitive to the assumptions on (*f*,*p*), and hence can be used to match with real admission count and to determine the credible range for *γ*_2_. There were around 15,711 patients admitted in total in Fangcang shelter hospitals [13] and around 2,500 operational beds in the two newly constructed emergency specialty field hospitals. However, due to the lack of knowledge about the admissions in other hospitals during the specific period, the *γ*_2_ value cannot be identified without further information.

### Intervention efficacy

**Fig. 6:**
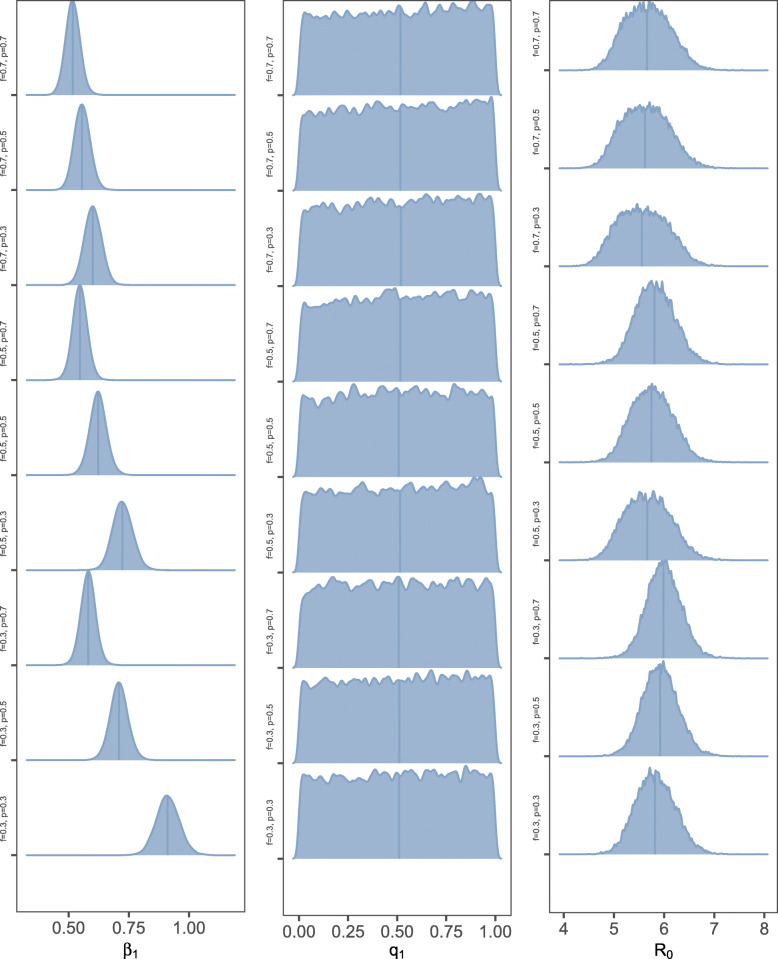
Posterior distributions of fitted parameters in Phase I under various scenarios. We performed fittings in a total of 81 scenarios about different assumed pairs of (*f*,*p*) for Phase I and selected 9 scenarios for *f*,*p*=0.3,0.5,0.7 in the presentation. The estimated values of transmission rate *β*_1_ are smaller given larger fraction of symptomatic individuals (*f*) or larger infectiousness of the asymptomatic individuals (*p*). The posterior distributions of the unobserved case fractions are independent from the assumed (*f*,*p*) pair

**Fig. 7:**
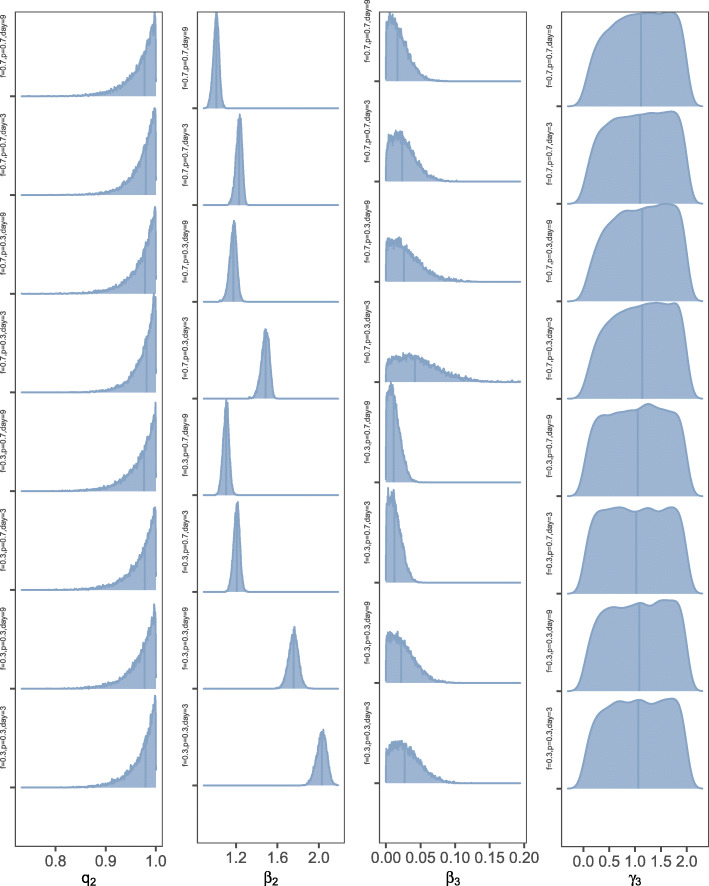
Posterior distributions of fitted parameters in Phase II under various scenarios. We performed fittings in a total of 324 scenarios for Phase II with various assumed sets of (*f*,*p*,*γ*_2_). Here we selected 9 scenarios with *f*,*p*=0.3 and *γ*_2_=1/3,1/9. The estimated values of *q*_2_,*β*_3_ and *γ*_3_ were independent from the set of parameters chosen, while the value of *β*_2_ increases as *γ*_2_ increases and depends on (*f*,*p*) similarly as in Phase I

The fitted parameters were quantifications of the average strengths of intervention strategies during multiple stages: *β*_1_,*β*_2_,*β*_3_ reflect the overall efficacy of almost no intervention, social distancing, and stay-at-home policies before lockdown, between Jan 24 to Feb 1, 2020, and after Feb 2, 2020. Among all 324 scenarios, the posterior distribution of *β*_3_ always falls in a very narrow interval close to zero (Figs. 6 and 7), meaning that there was extremely limited transmission effective contacts after the enforcement of stay-at-home policy. On the other hand, the transmission rate shortly after lockdown was shown to be around twice of that before the lockdown in all scenarios (Fig. 3d). Thus there were more transmission effective contacts between the susceptible and infectious individuals during the social distancing period compared with no intervention period. Such findings, however, are not hard to comprehend: right after the unprecedented lockdown, many people with suspected symptoms rushed to hospitals, waited hours in mixed crowds before seeing a doctor, getting tests, and obtaining medications. These extremely mixed crowds indeed posed increased effective contacts between infected individuals and susceptible people (including both susceptible patients and health care workers) and the medical system in Wuhan during that period was completely overwhelmed and as a result many patients had to go home even they were clinically diagnosed. Consequently, family cluster and community cluster infections increased dramatically during this special period [15, 16, 47].

The average fractions of identifiable symptomatic cases before lockdown (*q*_1_) and between Jan 24 to Feb 17, 2020 (*q*_2_) turned out to differ significantly: the posterior distribution of *q*_1_ was flat with mean around 0.5 under all scenarios, indicating that a considerable portion of symptomatic cases might have been missed before the increase of public awareness of the lockdown; while the posterior sampling of *q*_2_ was concentrated in narrow intervals close to 1.0 under all scenarios (Fig. 3e), meaning that cases with symptom onsets after the lockdown had been widely identified and documented. The last parameter to be fitted to real data was *γ*_3_ - the average rate of isolation after Feb 6, 2020, and not surprisingly this rate was estimated to be even slightly larger than 1.0. This means that all cases were promptly isolated upon their development of infectiousness after the centralized quarantine policy was put in effect and with the aids of the newly added medical resources, isolation and quarantine facilities.

### Infections in different phases

We calculated the number of infection incidences during phases from Jan 24 to Feb 1, 2020 (9 days), from Feb 2 to Feb 6, 2020 (5 days), from Feb 7 to Feb 17, 2020 (11 days), and after Feb 18, 2020. The relative numbers of infections during the four phases were found to be insensitive with respect to the assumptions on *γ*_2_ and *p*, but their actual values were clearly sensitive to the assumptions on *f* - which is intuitively understandable as the higher the fraction of asymptomatic cases is, the more infections could have been generated. We presented the scenario with *f*=0.9 (when 90% of the cases were symptomatic) in Fig. 3c. The daily infection incidences for symptomatic cases were not sensitive to *f* and thus similar in all scenarios: 3,780 (95% CI [3265,4246]) new cases per day from Jan 24 to Feb 1, 2020; 2,554 (95% CI [2203,3022]) per day from Feb 2 to Feb 6, 2020; and 206 (95% CI [15,508]) per day from Feb 7 to Feb 17, 2020. Our results indicated that Jan 24 to Feb 1, 2020 (right after lockdown) was the most severe period of the outbreak with 67.6*%* (95% CI [0.584,0.759]) detectable infections occurred during these 9 days. Although the transmission rate could have been reduced significantly after Feb 2, 2020, but as there were so many infectious cases in the community the transmission was still critical. New infection incidences were significantly brought down after the improvement of medical resources, thus both the stay-at-home order and the quick isolation of infectious individuals played the most essential roles in the containment of the outbreak.

### Asymptomatic, undetected and total cases

Other hidden quantities that can be estimated via our model would be the numbers of asymptomatic and undetected cases and the overall antibody prevalence level after the outbreak. Specifically, with a well-parameterized model, the total number of asymptomatic and undetected cases residing in Wuhan can be calculated via $\int _{0}^{\infty } \mu (A(t)+I_{2}(t))dt$, the total number of infected cases can be evaluated by $\int _{0}^{\infty } [\mu (A(t)+I_{2}(t))+\gamma (t)I_{1}(t)]dt$, and the overall antibody prevalence level can be estimated via $\int _{0}^{\infty } [\mu (A(t)+I_{2}(t))+\gamma (t)I_{1}(t)]dt/N_{2}$.

**Fig. 8:**
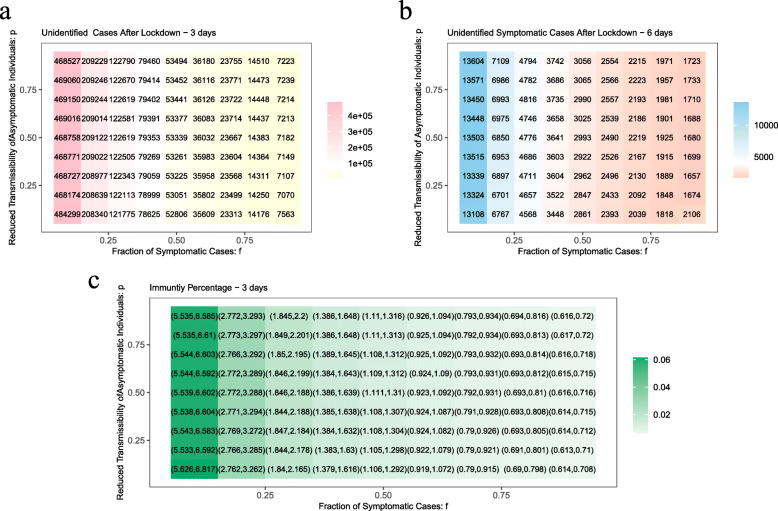
Undetected cases under various assumptions of the fraction of symptomatic cases (*f*) and reduced transmissibility of asymptotic individuals (*p*). **a** Possible total number of undetected cases (including asymptomatic and symptomatic) with recovery/removal dates after lockdown. **b** Possible total number of undetected symptomatic cases with recovery/removal dates after lockdown. **c** Possible percentage (95% CI) of population-wide antibody prevalence level after the outbreak. The number shown in each cell corresponds to the projected value from the model with each combination of (*f*,*p*) while $\gamma _{2}^{-1}=3\,\text {days}$

Recently, it was reported that for asymptomatic individuals the median duration of viral shedding was much longer and the virus-specific IgG levels were significantly lower compared to the symptomatic cases [42]. However, there are very few studies on the percentage of asymptomatic cases in the total infected population and their transmission ability [35, 41, 48]. First of all, we found these outcomes insensitive to the assumption on *γ*_2_: that is, under fixed *f* and *p* values, we obtained similar estimations on the total number of asymptomatic and undetected cases and overall antibody prevalence level regardless of the fixed value of *γ*_2_. This enabled us to present our estimations as a table based on assumptions about asymptomatic individuals. In Fig. 8a and b, we plotted the median values of the simulated total number of unidentified cases, respectively, including and excluding the asymptomatic individuals who were infected after lockdown. Clearly, the total number of asymptomatic and undetected cases depends positively on the fraction of asymptomatic cases and the transmissibility of asymptomatic individuals. In Fig. 8c, we plotted the 95% confidence intervals of our model estimations on the overall antibody prevalence in Wuhan for all possible scenarios with fraction of symptomatic cases *f* varies from 0.1 to 0.9 (i.e. the percentage of asymptomatic cases varies from 90% to 10%) and the reduced transmission ability *p* of asymptomatic individuals changes (to that of symptomatic ones) from 10% to 90%. Figure 8c indicates that at most 5∼6*%* of the whole population had contracted the virus.

To estimate the numbers of asymptomatic and undetected cases and the overall antibody prevalence level in Wuhan, we estimated *f* and *p* from the literature. Based on a total of 2,147 close contacts of COVID-19 132 cases in Ningbo, China, analysis of nucleic acid tests showed that 17% of cases were asymptomatic among all nucleic acid test-positive cases [39]. This agreed with the estimate from the 391 SARS-CoV-2 cases and 1,286 close contacts in Shenzhen, China, where approximately 20% of nucleic acid test-positive cases were asymptomatic [40]. A living systematic review and meta-analysis of 94 studies also showed that the overall estimate of the proportion of people who become infected with SARS-CoV-2 and remain asymptomatic throughout infection was 20% (95% CI [17%, 25%]) [49]. So we chose *f*=0.8. It was estimated that the infection rates of symptomatic and asymptomatic individuals were 6.30% and 4.11%, respectively [39]; that is, the transmissibility of asymptomatic individuals is about 70% of the symptomatic ones. So we set *p*=0.7 [35, 39–41]. From Fig. 8b and c, we estimated that there were 14,448 asymptomatic and undetected cases (95% CI [12,364,23,254]), which yields an estimate of a total of 64,454 infected cases (95% CI [62,370,73,260]) in Wuhan by March 31, 2020. Thus, the overall antibody prevalence level in the population of Wuhan was 0.745% (95% CI [0.693*%*,0.814*%*]) by the end of March 2020. Hence, due to the efficient containment strategies implemented, the population in Wuhan is far away from building up herd immunity [50, 51].

## Discussion

Wuhan is the capital of Hubei Province and is considered the political, economic, financial, commercial, cultural and educational center of Central China. There are more than one million undergraduate and graduate students from all around the world currently attending about 40 universities in the city. There were direct flights from Wuhan to most major international cities. The “Golden Waterway” of the Yangtze River and its largest tributary, the Han River, divide Wuhan into three districts: Hankou, Hanyang and Wuchang. It is a major transportation hub with dozens of railways and expressways passing through the city and connecting to other major cities. The infrastructure has been dramatically improved in China in the last 20 years by the fast development and expansion of the fast train and highway systems, which makes travel easier and faster. By taking the fast train from Wuhan, one can reach Shanghai in the east, Guangzhou in the south, Xian in the west and Beijing in the north within five hours. The ease of transportation can be a double-edged sword, when these advantages turned around and hurted in the case of the COVID-19 outbreak, that was first identified there. As a matter of fact, by the end of January 2020, COVID-19 had been spread from Wuhan to all other prefectures in Hubei Province, all other provinces, autonomous regions, municipalities, and special administrative regions in China as well as more than two dozen other countries.

Prevention and control strategies, such as early diagnosis and treatment of infected individuals, tracing and quarantining of exposed individuals, and isolation of infectious individuals, are standard and textbook-style measures for any infectious disease in human population. But how to interpret and implement these strategies, and more importantly, when to implement these measures is challenging for each different infectious disease, in particular for a disease caused by a coronavirus such as SARS-CoV, MERS-CoV, and this SARS-CoV-2. From the news and reports it seems that the local authorities in Wuhan did follow these measures in early January 2020 during the initial stage of the outbreak after 27 cases were reported on December 31, 2019, in which all these cases were associated with a seafood and wild animal market. The fact that 14 more patients who were not associated with the market were confirmed on January 10, 2020 strongly indicated that this virus can be spread from human to human. From the news and reports it seems that the public did not receive the complete information about the infectiousness and seriousness of the novel coronavirus in the early stage. Also, there were no more new cases reported in the next five days till January 16, 2020 (Fig. 1).

To prevent the geographic transmission and control local spread of infectious diseases, lockdown of infected regions is an easy-to-say but hard-to-implement policy for the potentially political, economical, social, epidemiological and other consequences, let alone the size, scale, and population of Wuhan. Nevertheless, on January 23, 2020 (two days before the Chinese Spring Festival), the local authorities locked down the entire Wuhan City and suspended all local and long-distance public transportation. People were requested to stay at home and wear face masks in public mandatorily. It is known that to control local transmission of infectious diseases, reducing the transmission rate and quarantining the exposed individuals are very effective measures. The transmission rate can be interpreted as the production of *c* (the contact rate between infectious and susceptible individuals) and *l* (the probability of infection per contact). Staying at home would help diminish daily contacts (*c*) and wearing face masks in public would help decrease the probability of virus transmission during contacts (*l*). From the point of view of prevention and control, locking down the entire city is the most effective way to prevent further spread of the virus to other regions and to reduce local transmission of the disease within the city. However, in the case of lockdown in Wuhan, nobody was prepared for such a large-scale lockdown. Many people with suspect symptoms ruched to hospitals, waited hours after hours in mixed crowds before seeing doctors, getting tests, and obtaining medicines, which created extremely mixed crowds of SARS-CoV-2 infected individuals and others and made some of those susceptible people more likely to be infected with the virus. Doctors and health care workers were overwhelmingly treating the large number of patients and some of them were infected. The labs were extremely short of the test kits. Hospitals were urgently short of beds. Many patients had to go home even they were clinically diagnosed. As a result, family cluster and community cluster infections increased dramatically in the first two week after lockdown [47]. Our estimations indicated that the transmission rate between Jan 24 and Feb 1, 2020 was on average twice as large as that before the lockdown and two-thirds of detectable infections occurred during this severe period. However, we would like to emphasize that this was caused by a mixture of the benefits of social distancing and the setbacks of the overwhelmed medical system and should never be interpreted as lockdown and social distancing being ineffective in slowing down the spread.

To overcome these difficulties, the local authorities had taken steps to face the reality. The number of labs that can perform RT-PCR tests was increased from 2 (before January 24) to 40 (after February 24) and the number of RT-PCR test kits was increased from 200 (before January 24) to 7,000 (after February 4). Two new specific hospitals, Huoshenshan with 1,000 beds and Leishenshan with 1,600 beds, were built in days and started to admit patients on February 4 and 8, respectively. Eleven sport centers, exhibition halls, and university dorms were turned into makeshift hospitals with more than 10,000 beds for confirmed patients with mild symptoms (Fig. 1). The 35,000 plus medical workers came from across China during the outbreak really helped the local medical system to treat the infected patients, while these nonpharmaceutical interventions were the key to control the COVID-19 outbreak in Wuhan in less than three months.

The complete outbreak data of Wuhan with 50,006 reported cases is notoriously hard to fit due to the sudden spike of cases on Feb 12, 2020 (Fig. 1), which is widely believed to be caused by the delay of case detection and reporting [15]. In our model, we incorporated the case detection dynamics and parameterized the delayed reporting rate accordingly in different phases, so that we can reconstruct the full transmission dynamics via data fitting. Indeed, using daily symptom onset data would be the best way to avoid errors in parameter estimates and model-based forecasts [16]. but such information was not available for the 17,365 clinically diagnosed cases [15]. Therefore, we used cumulative reported case data and considered the case detection dynamics to fulfill our needs of using the complete data and obtaining credible fitting outcomes. Also, our estimates of undetected and asymptomatic cases and the overall antibody prevalence level in the population of Wuhan were based on the estimates on the percentage of asymptomatic cases among all nucleic acid test-positive cases (1−*f*) and their transmission ability (*p*) [39, 40]. Further survey on the seroprevalence of SARS-CoV-2 antibodies in the population of Wuhan is needed which might show higher 1−*f* and *p* values, while the estimated undetected cases, asymptomatic cases, and the overall antibody prevalence level can still be obtained from Fig. 8c.

Since the COVID-19 outbreak in Wuhan was the first of its kind and there was very limited knowledge about the novel coronavirus, as well as the treatment and mitigation of its infection, very restrictive control and prevention measures were adapted and the outbreak was brought under control in a relatively short time. Compared to the outbreaks in some other regions, it seems that if lockdowns are lifted too early, the novel coronavirus will re-emerge and further lockdowns are needed. Our model and techniques can be modified to study the epidemics in other regions that have been experiencing multi-peaked and long-time outbreaks by using multi-step functions to estimate the model parameters and by employing multi-stage models to calibrate the much longer term data.

One quantity that we avoided to discuss is the basic reproductive number, *R*_0_, which was reported in almost all modeling works on COVID-19 and has been compared from study to study. *R*_0_ is defined as the average number of secondary infections that could be generated by one infectious individual over his/her entire illness period given a fully susceptible population. In practical ODE models, *R*_0_ is formulated by calculating the spectral radius of the next generation matrix obtained from the linearized system at the disease-free state, and serves as a threshold to tell whether or not the infectious population would increase in a certain time period. The actual value of *R*_0_ is significantly dependent on the model and the assumed parameter values, therefore, they might not be comparable if obtained from different models or under different parameterizations. Occasionally, *R*_0_ is erroneously compared with the daily (instantaneous) reproductive number in the early stage of the outbreak that is obtained by statistical methods, causing further confusions in understanding the infectiousness of an epidemic disease. Overall, the main purpose of calculating *R*_0_ and the effective reproductive number *R*_*e*_ is to evaluate the effectiveness of intervention strategies during various time periods. Instead, ODE modelers can easily calculate the actual number of infections at any time point from the well calibrated model (as we did in this study), and this can provide another straightforward approach to address the same question.

Our study has several limitations. Our results were based on assumed values of fixed model parameters and the assumed first day of transmission. Reasonable perturbations of the fixed model parameter values would not significantly alter our quantitative outcomes, however, alternative assumptions on the initial date of transmission would be worth further investigating. The initial date of transmission (day 0) is crucial in the setup of initial conditions for an ordinary differential equation (ODE) system and would impact the estimations of the outbreak growth rate in the early stage and thereafter. As the epicenter of the novel disease outbreak, it could take a long time for the scientific community to identify the origin of the virus and the time of the first human-to-human transmission in Wuhan. With limited information, we initiated our simulations on Dec 8, 2019, which is believed to be the earliest symptom onset date of all identified cases [3, 15, 16]. Further, multivariate data (such as the daily count of deaths and hospitalizations that are available in many other affected countries) would help with the enhancement of parameter estimations, which were hard to be collected for Wuhan given the overwhelmed public health system. A third aspect is that the age of the host plays a crucial role in the infection, transmission and mortality of COVID-19 [5, 15, 52, 53], which should be considered in future modeling studies.

## Conclusion

In the early stage of the COVID-19 outbreak, Wuhan experienced serious shortages of medical resources, long delays in case detection and reporting, and other issues. The outbreak was under control by the implementation of a series of nonpharmaceutical interventions (NPIs) including unprecedented lockdown of the city. A time-dependent compartmental model was developed to describe the dynamics of disease transmission and case detection across different periods determined by key events and interventions based on 50,006 reported cases and to estimate the number of asymptomatic and undetected cases. These results indicate that the combination of NPIs has successfully mitigated the outbreak in Wuhan and provide insights for designing control strategies and planing vaccination programs for other affected countries and regions.

## Data Availability

The datasets used and/or analyzed during the current study are available from the corresponding author on reasonable request. Declarations

## Abbreviations

CI: Confidence interval
COVID-19: Coronavirus Disease in 2019
elpd _loo_: Bayesian LOO estimate of the expected log pointwise predictive density
HCHP: Health Commission of Hubei Province
LOO package: leave-one-out cross-validation package
MCMC method: Monte Carlo Markov Chain method
NHCC: National Health Commission of China
NSNCD: National Stationary Notifiable Communicable Diseases
NPIs: nonpharmaceutical interventions
PPEs: personal protective equipments
PHEIC: public health emergency of international concern
RT-PCR test: reverse transcription polymerase chain reaction test
SARS-CoV-2: Severe Acute Respiratory Syndrome Coronavirus 2
WHO: World Health Organization
WMHC: Wuhan Municipal Health Commission
